# Conditional deletion of *Des1* in the mouse retina does not impair the visual cycle in cones

**DOI:** 10.1096/fj.201802493R

**Published:** 2019-01-15

**Authors:** Philip D. Kiser, Alexander V. Kolesnikov, Jianying Z. Kiser, Zhiqian Dong, Bhagirath Chaurasia, Liping Wang, Scott A. Summers, Thanh Hoang, Seth Blackshaw, Neal S. Peachey, Vladimir J. Kefalov, Krzysztof Palczewski

**Affiliations:** * Research Service, Louis Stokes Cleveland Veterans Affairs Medical Center, Cleveland, Ohio, USA;; † Department of Pharmacology, Case Western Reserve University School of Medicine, Cleveland, Ohio, USA;; ‡ Department of Ophthalmology and Visual Sciences, Washington University School of Medicine, St. Louis, Missouri, USA;; § Polgenix Incorporated, Cleveland, Ohio, USA;; ¶ Department of Nutrition and Integrative Physiology (NUIP), University of Utah, Salt Lake City, Utah, USA;; ‖ Diabetes and Metabolism Research Center, University of Utah, Salt Lake City, Utah, USA;; # Solomon H. Snyder Department of Neuroscience, Johns Hopkins University School of Medicine, Baltimore, Maryland, USA;; ** Cole Eye Institute, Cleveland Clinic, Cleveland, Ohio, USA;; †† Department of Ophthalmology, Gavin Herbert Eye Institute, University of California–Irvine, School of Medicine, Irvine, California, USA

**Keywords:** retinoid cycle, sphingolipidδ(4) desaturase, *Degs1*, photoreceptor, Müller glia

## Abstract

Cone photoreceptors are essential for vision under moderate to high illuminance and allow color discrimination. Their fast dark adaptation rate and resistance to saturation are believed to depend in part on an intraretinal visual cycle that supplies 11-*cis*-retinaldehyde to cone opsins. Candidate enzymes of this pathway have been reported, but their physiologic contribution to cone photoresponses remains unknown. Here, we evaluate the role of a candidate retinol isomerase of this pathway, sphingolipid δ4 desaturase 1 (Des1). Single-cell RNA sequencing analysis revealed *Des1* expression not only in Müller glia but also throughout the retina and in the retinal pigment epithelium. We assessed cone functional dependence on Müller cell–expressed *Des1* through a conditional knockout approach. Floxed *Des1* mice, on a guanine nucleotide-binding protein subunit α transducin 1 knockout (*Gnat1^−/−^*) background to allow isolated recording of cone-driven photoresponses, were bred with platelet-derived growth factor receptor α (Pdgfrα)-Cre mice to delete *Des1* in Müller cells. Conditional knockout of *Des1* expression, as shown by tissue-selective *Des1* gene recombination and reduced Des1 catalytic activity, caused no gross changes in the retinal structure and had no effect on cone sensitivity or dark adaptation but did slightly accelerate the rate of cone phototransduction termination. These results indicate that Des1 expression in Müller cells is not required for cone visual pigment regeneration in the mouse.—Kiser, P. D., Kolesnikov, A.V., Kiser, J. Z., Dong, Z., Chaurasia, B., Wang, L., Summers, S. A., Hoang, T., Blackshaw, S., Peachey, N. S., Kefalov, V. J., Palczewski, K. Conditional deletion of *Des1* in the mouse retina does not impair the visual cycle in cones.

The vertebrate retina contains 2 types of photoreceptor cells responsible for initiating vision: rods, which are exquisitely sensitive to dim light but desensitize rapidly with increasing illumination, and cones, which mediate color vision and are less light sensitive but remain active under high illuminance (1). The mechanism by which both cell types detect photons involves proteins known as opsins that utilize a vitamin A–derived chromophore, 11-*cis*-retinaldehyde. Absorption of light by the chromophore of these visual pigments effects a *cis-trans* isomerization, triggering protein conformational changes that initiate phototransduction (2, 3). This process also results in dissociation of all-*trans*-retinaldehyde from opsin, necessitating pathways for 11-*cis*-retinaldehyde renewal.

The classic visual cycle is one such regeneration pathway that has been known for more than a century and is biochemically and genetically well defined (4). This pathway is probably the sole mechanism for pigment regeneration in rods, and it also contributes to 11-*cis*-retinaldehyde production for cones (5–8). However, cone photoreceptor dark adaptation, which reflects the rate of visual pigment regeneration, is several times faster than that of rods, suggesting that cones have access to a privileged supply of visual chromophore (9). Support for a cone-specific intraretinal visual cycle involving Müller glia has been derived from biochemical (10, 11) and electrophysiologic (5, 12, 13) experiments, but genetic evidence for such an alternative visual cycle has, until recently, been lacking.

Studies in *Rlbp1^−/−^* mice have established that Müller glia–expressed cellular retinaldehyde-binding protein (Cralbp), an 11-*cis*-retinoid–binding protein, plays a pivotal role in rapid cone dark adaptation (14). Candidate enzymes of the alternative visual cycle pathway have been identified by using Müller cell cDNA library screening and candidate gene approaches. A putative isomerase of this pathway was identified by using the former approach as a protein known as sphingolipid δ4 desaturase 1 (Des1) (15). This enzyme, which was originally recognized as an essential enzyme of the *de novo* ceramide biosynthetic pathway (16), was suggested to catalyze the isomerization of retinol to form small quantities of 9-*cis* and 11-*cis*-retinol that could subsequently be trapped by binding to Cralbp or selective esterification (17, 18). Although adeno-associated virus-mediated expression of *Des1* in the Müller cells of mice lacking the classic visual cycle isomerase, retinal pigment epithelium (RPE)-specific 65 kDa protein (Rpe65), boosted cone electroretinogram (ERG) responses (15), the role of Des1 in physiologic cone pigment regeneration is currently unknown. Here, we address this question through the generation and characterization of Müller cell–conditional *Des1* knockout mice.

## MATERIALS AND METHODS

### Animal welfare

All experimental protocols were in accordance with the *Guide for the Care and Use of Laboratory Animals* (National Institutes of Health, Bethesda, MD, USA), were approved by the Louis Stokes Cleveland Veterans Affairs Medical Center, Case Western Reserve University, the Cleveland Clinic Cole Eye Institute, the Johns Hopkins University School of Medicine, and Washington University Institutional Animal Care and Use Committees. They conformed to the recommendations of the American Veterinary Medical Association Panel on Euthanasia and were conducted in accordance with the Association for Research in Vision and Ophthalmology Statement for the Use of Animals in Ophthalmic and Visual Research.

### Conditional *Des1* knockout animal generation and genotyping

Floxed *Des1* mice were generated by the Model Animal Research Center at Nanjing University, Nanjing, China. Exon 2 of the *Des1* gene (Degs1, NC_000067.6) was targeted for deletion because it encodes a majority of the protein sequence, and its removal introduces a change in the reading frame of the shortened mRNA. The targeting vector contained a FRT-flanked neomycin resistance marker located between exons 2 and 3 and a floxed exon 2. The vector was linearized and electroporated into 129S6/SvEvTac embryonic stem cells. Positive clones identified by using PCR and Southern blotting were microinjected into blastocysts to generate chimeras, which were then backcrossed to C57BL/6 mice to obtain heterozygous (*Des1^F/+^*) mice. Rederivation was conducted at the Case Western Reserve University Animal Resource Center Core Facility. *Des1^F/+^* mice were crossed with a germ-line FLP recombinase deleter strain (stock no. 009086; The Jackson Laboratory, Bar Harbor, ME, USA) to remove the neomycin cassette. The resulting mice were backcrossed to C57BL6/J mice (The Jackson Laboratory) to eliminate the FLPe recombinase gene from the genome. The presence and proper directionality of the exon 2-flanking loxP sites were confirmed with DNA sequencing. The resulting mice were then crossed with guanine nucleotide-binding protein subunit α transducin 1 (*Gnat1^−/−^*) mice (19) and mice expressing Cre recombinase under the platelet-derived growth factor receptor α promoter (Pdgfrα-Cre) (5) (stock no. 013148; The Jackson Laboratory) to produce Müller cell *Des1* conditional knockout mice lacking rod photoresponses. Mice were screened for the Rd8 allele as previously described (20) and were homozygous for the Met450 variant of Rpe65 (21). A liver-specific *Des1* conditional knockout mouse was also generated by crossing the floxed *Des1* mice with mice expressing Cre recombinase under the albumin promoter (Alb-Cre) (22) (stock no. 003574; The Jackson Laboratory). Primers and cycling parameters used for standard PCR-based genotyping of the *Des1*, *Gnat1*, Pdgfrα-Cre, and Alb-Cre loci are shown in Supplemental Table S1. PCR reactions were performed by using GoTaq Green Master Mix (Promega, Madison, WI, USA). Tissue-specific excision of *Des1* exon 2 by Cre recombinase was evaluated by using a multiplex PCR strategy (23) with primers and cycling parameters as described in Supplemental Table S1.

### Des1 activity assays

Des1 dihydroceramide desaturase activity was measured by using a radioactivity-based assay as previously described (5, 24). Mouse liver microsomes or whole retina homogenates, free of contaminating RPE, were used as enzyme sources in the assay. Assays were performed for 20 min at 37°C after a 10 min, 37°C preincubation period with NADH cofactor. The statistical significance of activity differences between experimental groups was assessed by ANOVA followed by application of the Holm-Sidak procedure to correct for multiple comparisons as implemented in Sigmaplot (Systat Software, San Jose, CA, USA).

### Optical coherence tomography

Retinas of mice aged 1–2 mo were analyzed *in vivo* with ultrahigh-resolution spectral domain optical coherence tomography (OCT) (Bioptigen, Morrisville, NC, USA) (25). Briefly, the mice were first administered a mydriatic eye drop (1% tropicamide) and then anesthetized with an intraperitoneal injection of ketamine (20 mg/ml) and xylazine (1.75 mg/ml) at a dose of 4 μl/g body weight. The A scan/B scan ratio was set at 1200 lines. Five OCT images scanned at 0 and 90° were acquired in the B mode, averaged, and saved as PDF files. Outer nuclear layer (ONL) thickness was measured 500 μm from the optic nerve head in the superior, inferior, temporal, and nasal retina. Values from each eye quadrant were averaged to give an overall value per eye.

### Dark- and light-adapted serial flash ERGs

Mice aged 2–4 mo were anesthetized with ketamine (80 mg/kg) and xylazine (16 mg/kg). Mydriatic eye drops (1% tropicamide; 2.5% phenylephrine) were used to dilate the pupils. ERGs were obtained by using a stainless steel active electrode that contacted the corneal surface through a layer of 1% carboxymethylcellulose. Needle electrodes placed in the cheek and tail provided reference and ground leads, respectively. Strobe stimuli were presented in an LKC Technologies (Gaithersburg, MD, USA) Ganzfeld device, either in darkness or superimposed on a steady adapting field (20 cd/m^2^) after at least 5 min of light adaptation. Responses were amplified, averaged, and stored by using an LKC UTAS E-3000 signal averaging system. Under dark-adapted conditions, stimulus strength ranged from −1.4 to 2.1 log cd·s/m^2^. The interstimulus interval was maintained at 2.1 s, and up to 20 consecutive responses were averaged for each stimulus condition.

After several days of recovery following the initial ERG measurements, mice were injected intraperitoneally with 50 μl of MB-001 in DMSO (160 μg/mouse). One hour later, mydriatic eye drops were instilled into the eyes to dilate the pupils, and the mice were exposed to a 10-min, 10,000 lux bleach. After a 4-h dark adaptation period, mice were anesthetized as described earlier, and a series of dark-adapted ERGs were recorded.

### *In vivo* cone photoreceptor dark adaptation measurements

Three-month-old mice were dark-adapted overnight and anesthetized with an intraperitoneal injection of a mixture of ketamine (100 mg/kg) and xylazine (20 mg/kg). Their pupils were dilated with a drop of 1% atropine sulfate applied to their eyes. The body temperature of the anesthetized mice was maintained at 37°C with a heating pad. ERG responses were recorded from both eyes by contact electrodes connected to the cornea by a drop of Gonak solution. Full-field ERGs were performed with a UTAS BigShot System (LKC Technologies) using Ganzfeld-derived test flashes of calibrated green 530 nm LED light, ranging in intensity from 0.24 to 3.74 cd·s/m^2^. Cone b-wave flash sensitivity (*S*_f_) was first determined in the dark, as *S*_f_ = *R*/(*R*_max_ × *I*), where R is the amplitude of cone b-wave dim flash response, *R*_max_ is the maximal response amplitude for that retina obtained with the brightest white light stimulus of the Xenon flash tube (700 cd·s/m^2^), and *I* is the flash strength. After measuring dark-adapted sensitivity, >90% of the M-cone visual pigment was bleached by a 30-s exposure to 520-nm LED light focused at the corneal surface. The bleaching fraction of cone pigment was estimated from the equation *F* = 1 − *e^−I⋅P⋅t^*, where *F* is the fraction of bleached pigment, *t* is the duration of the exposure to light (in seconds), *I* is the bleaching light intensity of unattenuated 520-nm LED light [∼1.3 × 10^8^ photons/(μm^2^·s)], and *P* is the photosensitivity of the mouse M-cone at the wavelength of peak absorbance (7.5 × 10^−9^ µm^2^), adopted from elsewhere (26). After the bleach, the recovery of cone b-wave sensitivity *S*_f_ was followed in the dark for up to 1 h. Mice were reanesthetized once (at 25 min after bleach) with a smaller dose of ketamine (approximately half the initial dose). At the same time, a 1:1 mixture of PBS and Gonak solutions was gently applied to the eyes to protect them from drying and to maintain electrode contacts. Data were analyzed by using the independent 2-tailed Student’s *t* test.

### *Ex vivo* cone photoreceptor dark adaptation measurements

Three-mo-old mice were dark-adapted overnight and euthanized by CO_2_ asphyxiation. The eyes were dissected, and a whole retina was removed from each eyecup under infrared illumination and stored in oxygenated aqueous L15 (13.6 mg/ml, pH 7.4) (MilliporeSigma, Burlington, MA, USA) solution containing 0.1% bovine serum albumin at room temperature. The retina was placed on filter paper with the photoreceptor side up and transferred to a perfusion chamber between 2 electrodes connected to a differential amplifier. The tissue was perfused with Locke’s solution containing 112.5 mM NaCl, 3.6 mM KCl, 2.4 mM MgCl_2_, 1.2 mM CaCl_2_, 10 mM HEPES, pH 7.4, 20 mM NaHCO_3_, 3 mM sodium succinate, 0.5 mM sodium glutamate, 0.02 mM EDTA, and 10 mM glucose. This solution was supplemented with 2 mM l-glutamate and 10 µM DL-2-amino-4-phosphonobutyric acid to block postsynaptic components of the photoresponse (27) and with 20 µM BaCl_2_ to suppress the slow glial PIII component (28). The perfusion solution was continuously bubbled with a mixture of 95% O_2_ and 5% CO_2_ and heated to 36–37°C.

Light stimulation consisted of 20-ms test flashes of calibrated 505-nm LED light controlled by a computer. The recovery of cone a-wave flash sensitivity (*S*_f_, defined as for the b-wave earlier) during dark adaptation was monitored by periodic test flashes after >90% of M-cone pigment was bleached with a 3-s exposure to 505-nm light. Photoresponses were amplified by using a differential amplifier (DP-311; Warner Instruments, Hamden, CT, USA), low-pass filtered at 30 Hz (8-pole Bessel), digitized at 1 kHz, and stored on a computer. Data were analyzed with Clampfit 10.4 (Molecular Devices, San Jose, CA, USA) and Origin 8.5 (OriginLab, Northampton, MA, USA) software using the independent 2-tailed Student’s *t* test.

### Single-cell RNA sequencing analysis

Adult male CD-1 mice used for droplet-based single-cell RNA sequencing (RNAseq) were purchased from Charles River Laboratories (Wilmington, MA, USA) and housed in a climate-controlled pathogen-free facility on a 14–10-h light/dark cycle. Animals were euthanized by cervical dislocation followed by decapitation, eyes were enucleated from the animals, and retinas dissected in fresh and cold 1 time phosphate-buffered saline. Retinas from 4 to 5 different animals were pooled to generate each sample used to ensure that appropriate numbers of cells were captured for downstream analyses. Dissected retinas were then transferred to 200 μl of cold HBSS per retina. An equivalent amount of papain solution [for 1 ml - 700 μl reagent grade water, 100 μl of freshly prepared 50 mM l-cysteine (MilliporeSigma), 100 μl of 10 mM EDTA, 10 μl of 60 mM 2-mercaptoethanol (MilliporeSigma), and 1 mg/ml papain (Worthington Biochemicals, Lakewood, NJ, USA)] was added and incubated at 37°C for 10 min, with slight trituration performed every 2 min. Six hundred microliters of Neurobasal Medium (Thermo Fisher Scientific) supplemented with 10% fetal bovine serum was added for every 400 μl of dissociation solution, and samples were further dissociated with gentle pipetting. Samples were subjected to DNAse treatment (5 μl RNAse free recombinant DNAseI; Roche, Basel, Switzerland) for every 1 ml of dissociation solution) for 5 min at 37°C. Cells were then pelleted through centrifugation for 5 min at 300 *g*. Liquid was carefully aspirated off the cell pellet, followed by resuspension of the pellet in 1–5 ml of Neurobasal medium with 1% fetal bovine serum, depending on required concentration of cells in suspension. Cellular aggregates were removed by straining cells through a 50 μm filter.

Single-cell suspensions for 10× libraries were loaded onto the 10× Genomics Chromium Single Cell (Pleasanton, CA, USA) system using the v.2 Chemistry per manufacturer’s instructions (29). Approximately 17,000 live cells were loaded per sample to capture transcripts from ∼10,000 cells. Estimations of cellular concentration and live cells in suspension was made through trypan blue staining and use of the Countess II Cell Counter (Thermo Fisher Scientific, Waltham, MA, USA). Single-cell RNA capture and library preparations were performed according to the manufacturer’s instructions. Sample libraries were sequenced on the NextSeq 500 (Illumina, San Diego, CA, USA).

Sequencing output was processed through the Cell Ranger 1.2.1 mkfastq (10× Genomics) and count pipelines by using default parameters. Reads were quantified by using the mouse reference index provided by 10× Genomics (refdata-cellranger-mm10 v.1.2.0). Raw count matrices for individual runs were manually aggregated, and cells were given unique, sample-specific cell identifiers to prevent duplication of nonunique barcodes across samples. The full raw count matrix was then used as input for the Monocle2 single-cell RNA-Seq framework (30).

A *t*-distributed stochastic neighbor embedding (*t*-SNE)-dimension reduction was performed on the top principal components learned from high variance genes. Mclust version 5.4 (31) was used to cluster cells in *t*-SNE-space, at which point cell type identity of clusters was assigned based on expression of known marker genes for either retinal or nonretinal tissue.

### Statistical analyses

Unless otherwise specified, data are presented as means ± the sem; *n* indicates the number of mice represented in each experimental group.

## RESULTS

### Single-cell RNAseq analysis of *Des1* expression in the adult mouse retina

**Figure 1:**
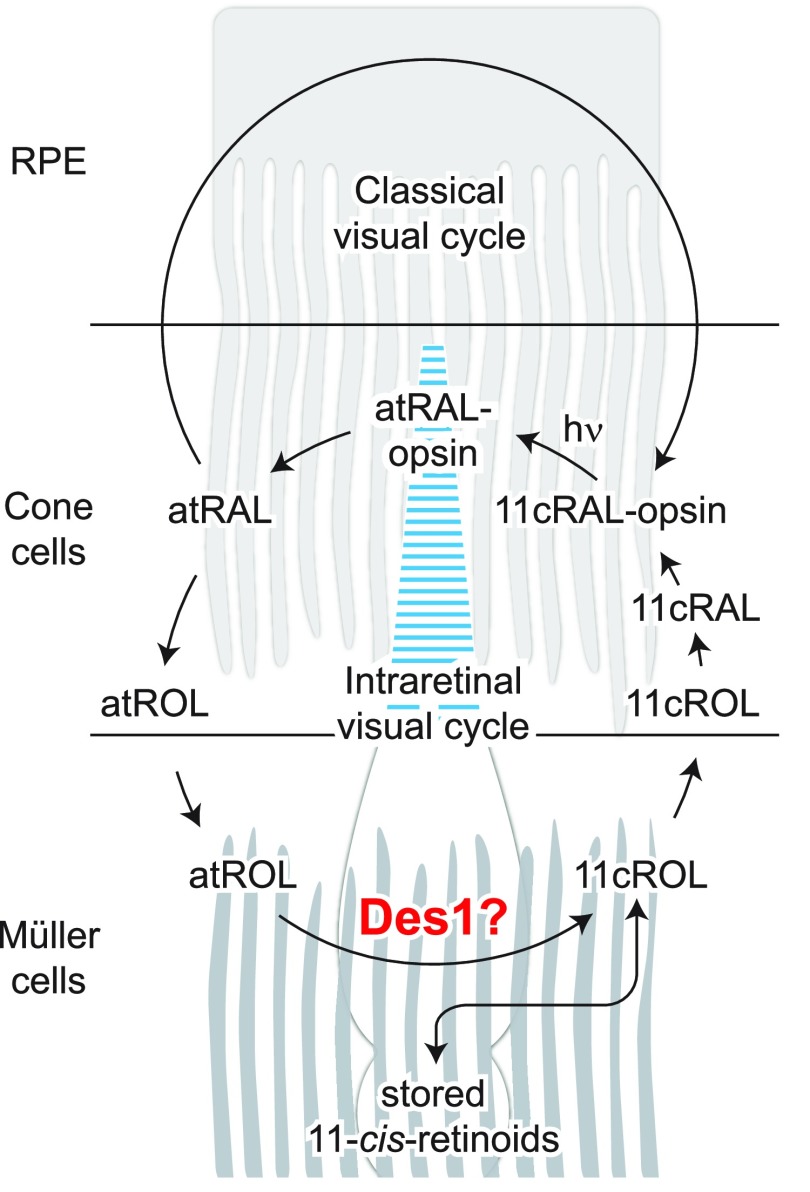
Pathways contributing to cone visual pigment regeneration in the mammalian retina. Cone pigment regeneration is mediated by both the classic visual cycle as well as an intraretinal visual cycle involving Müller glia. Des1, an enzyme originally recognized for its involvement in *de novo* ceramide production, is reported to catalyze the isomerization of retinol and has been proposed to be the physiologic isomerase of the cone-specific, intraretinal visual cycle. Here, we tested this proposal through the use of Müller cell *Des1* conditional knockout mice. Chemical intermediates of the classic visual cycle are omitted for simplicity.

**Figure 2:**
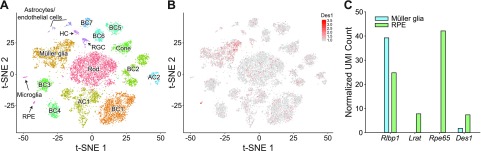
Single-cell RNAseq analysis of *Des1* expression in the adult mouse retina. *A*) Analysis of ∼17,000 cells revealed 17 different cell clusters from the adult whole mouse retina. *B*) *Des1* expression in the whole mouse retina at the single-cell level (red points). *C*) Comparative levels of *Des1*, *Rlbp1*, *Lrat1*, and *Rpe65* transcripts in the Müller glia and RPE cell clusters. AC, amacrine cell; BC, bipolar cell; HC, horizontal cell; RGC, retinal ganglion cell; UMI, unique molecular identifier.

*Des1* expression has been reported in Müller glia and the RPE (15), but its detailed distribution within the neural retina has not been described to date (**Fig. 1**). We therefore performed a single-cell RNAseq analysis of *Des1* expression in the adult mouse retina. RNAseq data were obtained from ∼17,000 individual retinal cells. The data were analyzed by *t*-SNE (32) resulting in cell clusters that were visualized on a two-dimensional plot and correspond to distinct retinal cell types (**Fig. 2*A***). The cell clusters were identified based on characteristic gene expression signatures (Supplemental Figs. S1–S3). The data set was generated from retinal cells obtained from 4 separate 2-mo-old mice to account for potential variability in *Des1* expression. As expected from published results, *Des1* transcripts were found in Müller glia from all 4 biologic replicates, with some differences in expression level between samples (Fig. 2*B* and Supplemental Fig. S4). However, *Des1* was not confined to Müller glia because *Des1*-positive cells were found in all of the cell clusters distinguished in this analysis, including rods, cones, RPE, bipolar cells, amacrine cells, horizontal cells, microglia, and the astrocyte/endothelial mixed cell cluster. Interestingly, *Des1* expression was highest in the RPE, and RPE levels were substantially above those in Müller glia (Fig. 2*C*). The RNAseq analysis allowed us to compare the expression level of *Des1* versus other genes involved in visual chromophore production (Fig. 2*C* and Supplemental Fig. S5). We found that *Des1* is expressed at much lower levels in either Müller cells or RPE compared with the expression level of *Rpe65* in the RPE. Moreover, the levels of *Rlbp1* transcripts in Müller glia and RPE greatly exceeded those of *Des1* in these tissues.

### Generation of *Des1* conditional knockout mice

**Figure 3:**
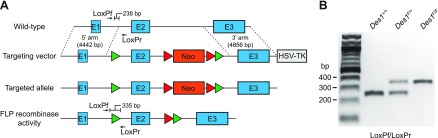
Generation and genotyping of floxed *Des1* mice. *A*) Exon 2 of the *Des1* gene was targeted for deletion by insertion of parallel flanking LoxP sequences. The targeting vector also contained a neomycin-resistance cassette, flanked by FLP recombinase targeting sequences, to allow for selection of successfully targeted embryonic stem cells. The neomycin cassette was subsequently removed by crossing floxed *Des1* mice with FRT germline deleter mice to yield mice lacking the neomycin cassette that were used for further breeding and analysis. The green and red triangles represent LoxP and FRT sequences, respectively. PCR primer binding sites and sizes of expected products are shown on the figure. Primers sequences are shown in Supplemental Table S1. HSV-TK, herpes simplex virus thymidylate kinase gene. *B*) PCR genotyping results for wild-type (*Des1^+/+^*), heterozygous (*Des1^F/+^*), and homozygous (*Des1^F/F^*) mice.

The *Des1* gene was targeted for inactivation by insertion of LoxP sites flanking exon 2 (**Fig. 3*A***). This exon contains a majority of the coding sequence, including regions encoding key catalytic residues, and its excision results in a frameshift, both of which ensured expression of nonfunctional Des1 following Cre-mediated recombination. The mice were generated according to standard procedures with the presence of the floxed *Des1* allele assessed by standard PCR-based genotyping as shown in Fig. 3*B*. *Des1^F/F^* mice were subsequently crossed with *Gnat1^−/−^* (19) Pdgfrα-Cre (33) mice to generate animals of the desired genotypes. Pdgfrα-Cre mice were developed specifically for conditional gene knockout in Müller cells and have been shown to be effective for this purpose in several studies, *e.g.* (34–36). Additionally, we crossed the *Des1^F/F^* mice with a transgenic mouse line that expresses Cre recombinase under the albumin promoter to achieve conditional knockout of *Des1* in the liver (37). These animals were used as controls to test the efficiency of Cre-mediated *Des1* exon 2 deletion, which can be readily verified as the liver microsome fraction is a source of robust Des1 activity (24). Mice of all genotypes investigated reproduced normally with offspring frequencies conforming to the expected Mendelian pattern. No consistent macroscopic phenotypic changes were observed for any of the genotypes we investigated.

### Deletion of *Des1* in the liver

**Figure 4:**
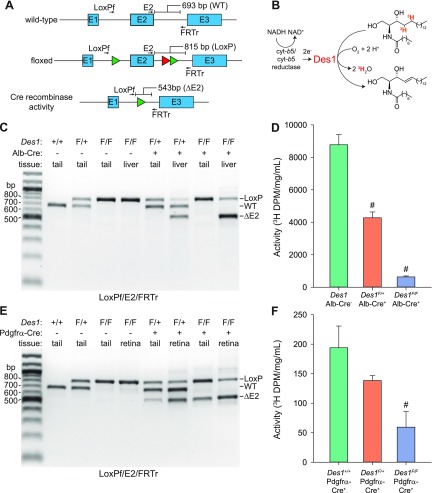
Genome- and protein-level analysis of efficiency and tissue specificity of *Des1* conditional knockout. *A*) Multiplex PCR strategy for detecting Cre recombinase activity directed toward the floxed *Des1* allele. The green and red triangles represent LoxP and FRT sequences, respectively. Primer binding positions are shown as arrows above and below the gene schematics. Primer sequences are given in Supplemental Table S1. *B*) Assay used to assess the level of Des1 activity in targeted tissues. Des1 activity against a tritiated dihydroceramide substrate releases tritiated water, which can be detected by scintillation counting. *C*) Multiplex PCR showing the susceptibility of *Des1* exon 2 deletion in wild-type or floxed *Des1* mice expressing Cre recombinase under the albumin promoter. Primers used for the reactions are listed below the gel image. *D*) Des1 activity assays conducted by using liver microsome samples from 1- to 2-mo-old mice as the enzyme source. Aqueous radioactivity produced during the reaction was normalized to the total protein concentration in the assay mixture. The “Des1 Alb-Cre” bar shows pooled data from Cre-negative *Des1^+/+^*, *Des1^F/+^*, and *Des1^F/F^* mice, which exhibited comparable activity levels. *Des1* Alb-Cre^−^, *n* = 3; *Des1^F/+^* Alb-Cre^+^, *n* = 3; *Des1^F/F^* Alb-Cre^+^, *n* = 4. *E*) Multiplex PCR showing the susceptibility of *Des1* exon 2 deletion in wild-type or floxed *Des1* mice expressing Cre recombinase under the *Pdgfrα* promoter. Primers used for the reactions are listed below the gel image. *F*) Des1 activity assays conducted by using whole retinal lysates, free of RPE contamination, from 1- to 2-mo-old mice of the indicated genotypes as the enzyme source. Aqueous radioactivity produced during the reaction was normalized to the total protein concentration in the assay mixture. *Des1^+/+^* Pdgfrα-Cre*^+^*, *n* = 2; *Des1^F/+^* Pdgfrα-Cre^+^, *n* = 10; *Des1^F/F^* Pdgfrα-Cre^+^, *n* = 4. Data (*D*, *F*) are presented as means ± sem. *^#^* *P* < 0.01 *vs.* Cre negative (*D*) or wild-type (*E*) *Des1* control groups.

We tested the efficiency of Cre-mediated inactivation of the *Des1* gene at both the gene and protein levels by using multiplex PCR (**Fig. 4*A***) and Des1 dihydroceramide desaturase activity assays (Fig. 4*B*). We validated the excisability of the LoxP-targeted exon 2 of *Des1* in *Des1* floxed mice expressing the Alb-Cre transgene. As shown in Fig. 4*C*, excision of *Des1* exon 2 occurred in the livers of *Des1^F/+^* and *Des1^F/F^* mice positive for the Alb-Cre transgene, whereas recombination was undetectable in all other samples. The PCR analysis suggested that *Des1* exon 2 excision was nearly quantitative, which is consistent with previous reports of gene recombination in the livers of Alb-Cre^+^ mice (22). A parallel loss of Des1 enzymatic activity was observed with *Des1^F/F^* Alb-Cre^+^ and *Des1^F/+^* Alb-Cre^+^ mice displaying ∼7 and 49% of the activity observed in mice with 2 functional copies of the *Des1* gene (Fig. 4*D*). These data showed the functionality of the *Des1* conditional knockout strategy.

### Deletion of *Des1* in the retina

We next examined the efficiency of *Des1* exon 2 excision in retina and tails of floxed *Des1 Gnat1^−/−^* mice expressing the Pdgfrα-Cre transgene. Care was taken to avoid contamination of the neural retina with RPE during the retinal tissue isolation procedure in light of the high level of *Des1* expression in the RPE (Fig. 2*C*). The multiplex PCR results in these mice were largely similar to what we observed in Alb-Cre transgenic mice with 2 notable differences (Fig. 4*E*). First, exon 2 excision was observed not only in retinal tissue of Pdgfrα-Cre*^+^* mice but also to a lesser extent in the tail samples from these mice. This result is not unexpected as *Pdgfrα* is known to be expressed in nonretinal cells such as adipocytes and other connective tissue cell types (38, 39). Second, the efficacy of *Des1* exon 2 excision by Cre in retinal tissue was somewhat lower than the deletion efficacy in the livers of Alb-Cre^+^ mice. This lower efficiency was reflected in the retinal Des1 activity results in which *Des1^F/F^* Pdgfrα-Cre*^+^* and *Des1^F/+^* Pdgfrα-Cre*^+^* mice displayed ∼31 and 72% of the activity associated with retinal samples from mice with 2 functional copies of the *Des1* gene (Fig. 4*F*). Notably, Des1 activity was ∼50 times lower in whole retinal cell lysates compared with liver microsomes.

We examined *Pdgfrα* transcripts in our single-cell RNAseq data set in an attempt to gauge the expression pattern of *Pdgfrα* promoter-driven Cre expression in the retina (Supplemental Fig. S1). Interestingly, *Pdgfrα* expression is not detectable in most neural retina cell types, with the astrocyte/endothelium cluster being the only one containing significant amounts of the transcript. Although surprising, this result is consistent with the original study describing Pdgfrα-Cre mice in which Müller cell expression of *Pdgfrα* was not detectable by microarray analysis (33). Despite this lack of native *Pdgfrα* mRNA expression, Cre activity specifically in Müller glia was clearly detected as shown by LacZ reporter mice. In light of the relatively broad expression of *Des1* in the retina (Fig. 2*A, B*) and the apparent confinement of retinal Cre activity in Pdgfrα-Cre mice to Müller glia, we can largely attribute the incomplete deletion of *Des1* and loss of Des1 activity in *Des1^F/F^ Gnat1^−/−^* Pdgfrα-Cre^+^ mice to remaining functional expression of Des1 in retinal cells other than Müller glia. Taken together with previous results from studies using the Pdgfrα-Cre mouse, our data provide strong evidence for substantial *Des1* conditional knockout in retinal Müller cells.

### *Des1* conditional knockout has no detectable effect on mouse retinal structure

**Figure 5:**
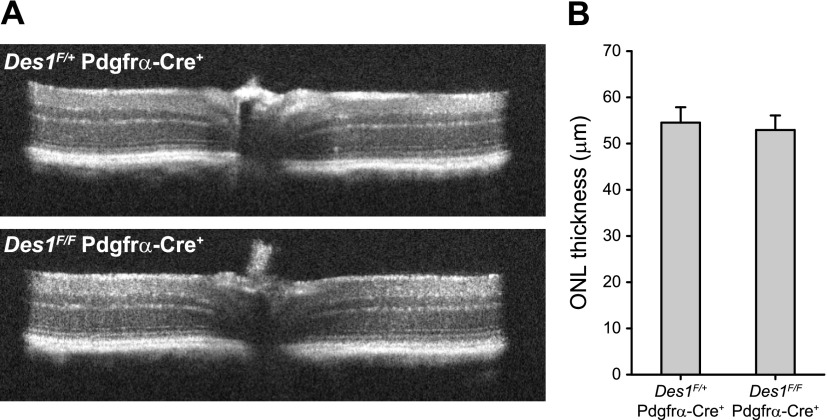
Analysis of *Des1* mouse retinal structure by OCT. *A*) Representative OCT images from *Des1^F/+^ Gnat1^−/−^* Pdgfrα-Cre^+^ (top) and *Des1^F/F^ Gnat1^−/−^* Pdgfrα-Cre^+^ (bottom) mice. The left and right portions of the images represent the inferior and superior regions of the retina, respectively. The bracket demarcates the ONL. *B*) Quantification of ONL thickness. The ONL measurements were made 500 μm from the optic nerve head in the superior, inferior, nasal, and temporal quadrants of the retina and then averaged. The data are presented as overall averages ± sem. *Des1^F/+^ Gnat1^−/−^* Pdgfrα-Cre^+^, *n* = 6; *Des1^F/F^ Gnat1^−/−^* Pdgfrα-Cre^+^, *n* = 8.

To assess whether *Des1* conditional deletion induces any major structural abnormalities in the retina, we assessed the morphology of the retina *in vivo* using OCT. As shown in **Fig. 5*A***, the characteristic reflectivity pattern of the retinal layers in *Des1^F/F^ Gnat1^−/−^* Pdgfrα-Cre^+^ mice was indistinguishable from the corresponding heterozygous mice expressing a functional copy of *Des1* and matched that of previously reported wild-type mice (40). Quantification of the retinal ONL thickness, which is a distinct layer of hyporeflectivity on OCT indicating the quantity of photoreceptor nuclei, also showed no differences between the conditional knockout mice and heterozygote controls within the error of the data (Fig. 5*B*).

### *Des1* conditional knockout does not affect the amplitude and sensitivity of cone photoreceptor responses

**Figure 6:**
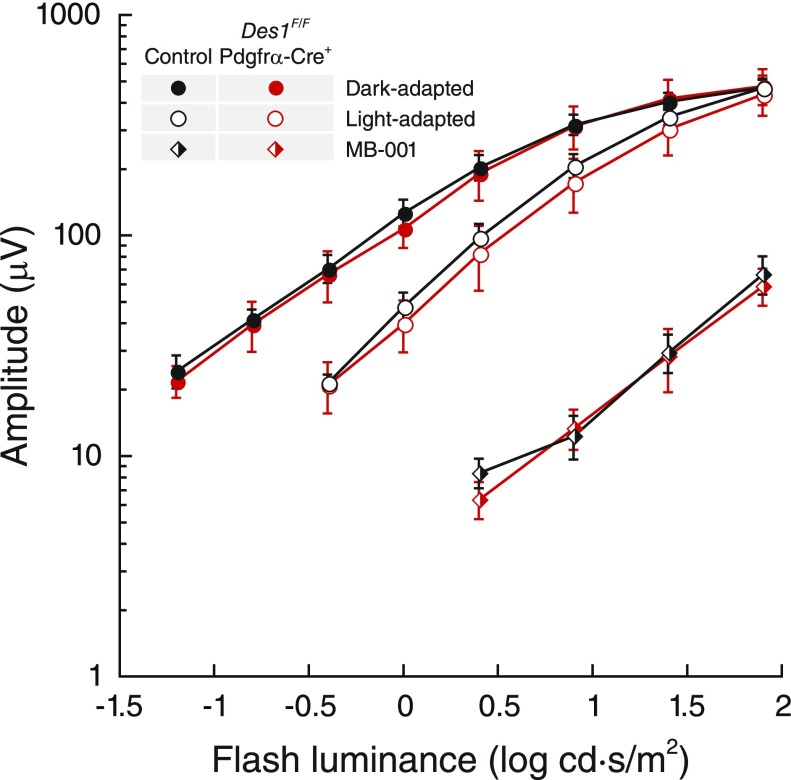
Impact of retinal *Des1* conditional deletion on single-flash ERG b-wave responses of murine cone photoreceptors. Full-field single-flash ERGs were recorded from 2- to 3-mo-old mice. The group labeled “control” was aggregated from littermates expressing at least 1 functional copy of *Des1*, all of which exhibited comparable ERG responses. After recovery from initial dark- and light-adapted ERG recordings mice were administered a single 160 μg dose of MB-001 by intraperitoneal injection and 2 h later administered mydriatic eye drops and then exposed to 10,000 lux illumination for 10 min followed by a 4 h dark adaptation period. Dark-adapted ERGs were then recorded from these mice. Data are shown as average ± sem. Control mouse group, *n* = 6; *Des1^F/F^ Gnat1^−/−^* Pdgfrα-Cre^+^ group, *n* = 4.

To assess whether diminished *Des1* expression impairs cone-driven photoresponses *in vivo*, ERGs were recorded in response to a series of light flashes from *Des1^F/F^ Gnat1^−/−^* Pdgfrα-Cre*^+^* mice and control mice containing a functional *Des1* allele under both dark- and light-adapted conditions. We tracked the b-wave amplitudes for these experiments, which is correlated with cone photoreceptor light responsiveness as opposed to smaller a-wave amplitudes, which have more complex electrical contributions including components from the inner retina (41). As shown in **Fig. 6**, dark-adapted cone responses were essentially identical between the 2 groups of mice. After exposure to low-intensity light for 5 min, ERG responses were recorded again to flashes superimposed upon the background. Under these conditions, a slight reduction in flash sensitivity was observed at midrange intensities but the differences between the two groups of mice were within the error of the data. In an attempt to tease out a potentially subtle ERG effect that could be masked by classic visual cycle–mediated cone pigment regeneration, dark-adapted cone ERG responses were also recorded from mice treated with the classic visual cycle inhibitor, MB-001 (5, 42), and then subjected to a strong photobleach so that cone pigment regeneration would rely exclusively on intraretinal visual cycle activity. As expected, cone responses for both groups of mice were substantially desensitized after the photobleach, consistent with the involvement of classic visual cycle activity in cone pigment regeneration. However, the residual responses of the 2 groups were not significantly different. Taken together, these ERG results do not support a role for Müller cell-expressed Des1 in the regeneration of cone visual pigments.

### *Des1* conditional knockout in the mouse retina does not affect cone photoreceptor dark adaptation

**Figure 7:**
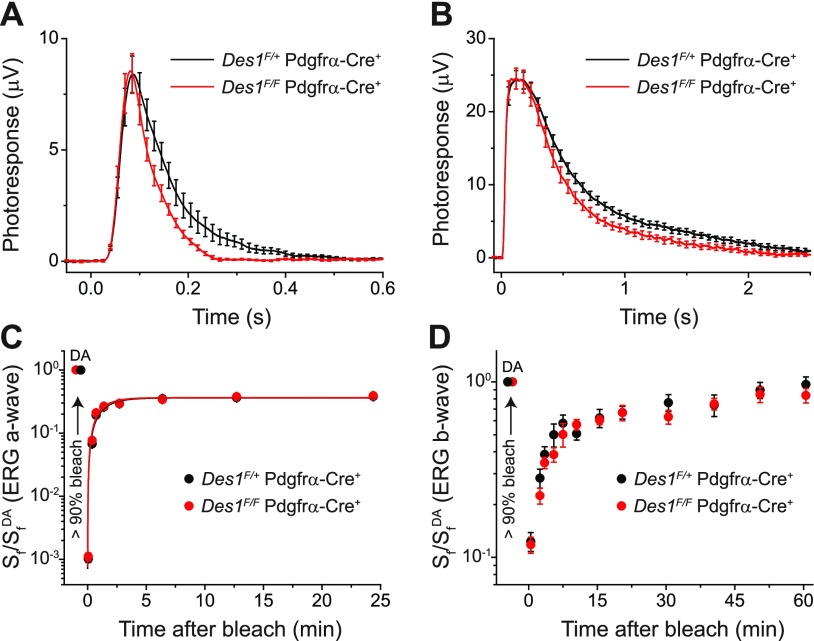
Physiologic characterization of cones in *Des1^F/+^ Gnat1^−/−^* Pdgfrα-Cre^+^ control and *Des1^F/F^ Gnat1^−/−^* Pdgfrα-Cre^+^ mutant mice and their dark adaptation. *A*) Kinetics of activation and inactivation of cone phototransduction in control and Des1-deficient mice measured by transretinal (*ex vivo*) ERG. Population-averaged (mean ± sem) dim flash responses to test stimuli of 7.0 × 10^3^ photons/μm^2^ (*n* = 13 for controls, and *n* = 10 for mutants). *B*) Comparison of saturated cone responses from control and Des1-deficient mice obtained by *ex vivo* ERG. Population-averaged (mean ± sem) responses to test stimuli of 2.0 × 10^6^ photons/μm^2^ (*n* = 13 for controls, and *n* = 10 for mutants). *C*) Recovery of cone ERG a-wave flash sensitivity (*S*_f_) in isolated retinas of control (*n* = 12) and Des1-deficient (*n* = 10) animals after bleaching >90% of cone pigment at time 0 with 505 nm LED light. Data were fitted with single-exponential functions that yielded the recovery time constants of 1.2 ± 0.2 and 1.1 ± 0.2 min, respectively. *S*_f_^DA^ denotes the sensitivity of dark-adapted cones. *D*) Recovery of photopic ERG b-wave *S*_f_ *in vivo* in control (*n* = 10) and Des1-deficient (*n* = 10) mice after bleaching >90% of cone pigment at time 0 with 520 nm LED light. *S*_f_^DA^ designates the sensitivity of dark-adapted cones.

To evaluate further the effect of conditional deletion of *Des1* on cone function, we performed transretinal (*ex vivo*) ERG recordings. This approach allowed us to pharmacologically isolate the photoreceptor component of the ERG response and quantify the effect of *Des1* deletion on cone function. Comparison of population-averaged cone dim flash responses in *Des1^F/+^ Gnat1^−/−^* Pdgfrα-Cre^+^ control and littermate *Des1^F/F^ Gnat1^−/−^* Pdgfrα-Cre^+^ knockout mice revealed no effect of *Des1* deletion on their amplitude (meaning comparable photosensitivities) and the rising phase of the response (meaning normal activation of phototransduction) in mutant mice. However, a slight acceleration of the response recovery in the cones from Des1-deficient retinas was observed (**Fig. 7*A***). Similar acceleration could be observed from saturated responses to brighter test flashes in mutant retinas, yet their maximal amplitudes were comparable to those of cones in control retinas (Fig. 7*B*). Thus, *Des1* conditional knockout in retinal Müller cells did not have a detrimental effect on cone function. Instead, it resulted in a mild acceleration of the inactivation of the cone phototransduction cascade.

To determine whether Des1 plays a role in the cone-specific intraretinal visual cycle, the kinetics of cone dark adaptation was examined both in the isolated retina by *ex vivo* ERG recordings, and in the intact eye by *in vivo* ERGs. As previously shown (43), cone pigment regeneration in the isolated retina is driven exclusively by the intraretinal visual cycle. Thus, suppression of this pathway by deleting *Des1* would be expected to delay or completely block cone pigment regeneration and dark adaptation in the isolated retina. However, we observed that both the kinetics of cone dark adaptation and the final level of recovery of cone sensitivity were identical in *Des1^F/+^ Gnat1^−/−^* Pdgfrα-Cre^+^ controls and littermate *Des1^F/F^ Gnat1^−/−^* Pdgfrα-Cre^+^ knockout retinas (Fig. 7*C*). Similarly, cone dark adaptation in the intact eye, driven by the combined action of the classic RPE visual cycle and the Müller cell–dependent retina visual cycle was also not affected by the deletion of *Des1* in the retina (Fig. 7*D*). Together, these results clearly show that although the targeted deletion of *Des1* in mouse Müller cells slightly accelerates the inactivation of cone phototransduction, it does not affect the regeneration of cone visual pigment and cone dark adaptation *via* the intraretinal visual cycle. As expected, the slower, RPE-driven (5) phase of cone dark adaptation (after 15 min) *in vivo* was not compromised in Des1-deficient mice.

## DISCUSSION

Cone photoreceptor function is supported by 2 visual chromophore sources, one being the classic visual cycle and the other a Müller cell–based pathway that is responsible for the characteristic rapid dark adaptation and saturation resistance of cones (9). Des1 was the first candidate enzyme of the Müller cell pathway to be suggested (15), but its physiologic role in cone pigment regeneration remained unexplored. The severely impaired viability or embryonic lethality associated with global *Des1* knockout (44) has been a barrier to the genetic study of this protein in mammalian retinal physiology. The pathology in these mice is due to blockade of the *de novo* ceramide biosynthetic pathway in which Des1 performs the key transformation of dihydroceramide into ceramide (45). The latter compound can act as a signaling molecule or be further metabolized into a variety of sphingolipids (46, 47). Interestingly, ceramide may play a role in apoptotic death of RPE (48), and inhibition of *de novo* ceramide biosynthesis has been identified as a therapeutic strategy for treatment of retinopathies (49–51). The high level of Des1 expression in the RPE that we observed suggests that this cell layer could be an important source of ceramide within the retina.

We have overcome the limitations associated with global *Des1* knockout in the present study through the use of Müller cell–conditional *Des1* knockout mice, which had no gross phenotypic defects or reproductive difficulties. We show that loss of Des1 activity in the retina does not result in retinal structure changes observable by OCT. By generating these mice on a *Gnat1^−/−^* background, we were able to show that Des1 is not obligatory for sustaining normal cone photoresponses and rapid dark adaptation. Interestingly, using *ex vivo* ERG measurement, we did observe that phototransduction termination is slightly more rapid in *Des1* conditional knockout mice compared with controls. Phototransduction processes involve a number of membrane-bound proteins whose function could be modified by the changes in membrane fluidity that are expected to accompany Des1 loss of function owing to perturbations in sphingolipid formation. Indeed, a sizable portion of retinal fatty acids are contained within sphingolipids (52). Alternatively, the change in phototransduction kinetics could be caused by subtle alterations in cone outer segment morphology or by changes in the expression of one or more of the cone phototransduction proteins. It also remains unresolved whether the functional change is due to loss of Des1 function in the targeted Müller cells or in one of the other Des1-expressing retinal cells types identified here through single-cell RNAseq analysis, including cones in which low-level Cre-expression might occur.

The dispensability of *Des1* expression in Müller glia for mouse cone pigment regeneration is consistent with previous pharmacologic studies that showed only mild changes in cone cell function upon treatment with high doses or concentrations of the Des1 inhibitor fenretinide (5). It is important to note that the experiments presented here were performed in mice, which are rod-dominant animals, whereas Des1 retinoid isomerase activity was identified by using a Müller cell library from chickens (15), which are cone dominant and may have independently adopted Des1 as a supplemental retinoid isomerase. However, previous results using fenretinide in ground squirrels indicate that Des1 activity is not required for rapid cone dark adaptation in cone-dominant mammals (5).

Considering the limited role for Müller cell-expressed Des1 in the mouse cone visual cycle indicated by our experiments, the question remains how 11-*cis*-retinoids that are present in Müller glia (53, 54) and are responsible for fast and specific regeneration of cone pigments (13, 14) are formed. Our previous estimates (5) suggest that 11-*cis*-retinoids found in complex with Cralbp within Müller cells alone could sustain cone photoreceptor function without the need to invoke alternative visual cycle enzymatic machinery. Although we cannot exclude the possibility that a yet-to-be-identified retinoid isomerase mediates such 11-*cis*-retinoid formation, it is remarkable that no gene mutations specifically affecting cone photoreceptor dark adaptation have been found in mice or humans, and efforts to purify isomerase II enzymes from the neural retina for molecular identification have not been successful. The anatomically intertwined nature of the RPE, photoreceptor, and Müller cell regions likely enables facile transfer of retinoids between these cell types, which contributes to the challenge of tracing the origin of 11-*cis*-retinoids in the neural retina. The lack of 11-*cis*-retinoids in the retinas of *Rpe65* knockout mice (6, 7) would suggest that 11-*cis*-retinoids in Müller glia are derived from classic visual cycle activity. Diminished *Des1* expression in the retinas of *Rpe65^−/−^* mice has been put forth as an alternative explanation for the extreme visual chromophore deficiency in these animals (15). However, the limited role of Müller cell-expressed Des1 in physiologic 11-*cis*-retinoid biosynthesis in mice indicates that an RPE-origin for the 11-*cis*-retinoids found within their Müller cells should be reconsidered.

## Supplementary Material

This article includes supplemental data. Please visit *http://www.fasebj.org* to obtain this information.

## Abbreviations

Cralbp: cellular retinaldehyde-binding protein
Des1: sphingolipid δ4 desaturase 1
ERG: electroretinogram
Gnat1: guanine nucleotide-binding protein subunit α transducin 1
OCT: optical coherence tomography
ONL: outer nuclear layer
Pdgfrα: platelet-derived growth factor receptor α
RNAseq: RNA sequencing
RPE: retinal pigment epithelium
Rpe65: retinal pigment epithelium-specific 65 kDa protein
*t*-SNE: *t*-distributed stochastic neighbor embedding
