# Supplementary material for: Conditional deletion of Des1 in the mouse retina does not impair the visual cycle in cones
Source: FASEB J. 2019 Jan 15;33(4):5782–92. doi: 10.1096/fj.201802493R (PMC6436658; doi:10.1096/fj.201802493R)
Supplement: Supplementary file 1 [file fj.201802493R.sd1.docx]

**SUPPLEMENTARY TABLES**

**Table S1 –** *Primers and cycling parameters used for genotyping and expected PCR product sizes*

| **Allele discrimination** | **Primers (5’ - 3’)** | **PCR cycling parameters** | **Reaction products** |
| --- | --- | --- | --- |
| Wild-type *Des1*, Floxed *Des1* | LoxPf:  GTGTCACGATGCCCAGGTGTT  LoxPr:  AGTGCTCTTGTGCCCTTAGCC | 1) 94 °C x 5 min  2) 94 °C x 45 sec  3) 58 °C x 30 sec  4) 72 °C x 1 min  5) 72 °C x 10 min  steps 2-4 repeated for 35 cycles | Wild-type*:*  238 bp  Floxed *Des1*: 335 bp |
| WT *Gnat1*, *Gnat1* KO | YF199: GAGGATTGGGAAGACAATAGCAG  YF207: CACCAGCACCATGTCGTAAG  YF148: CGAGTTCATTGCCATCATCTACG  YF149: ATACCCGAGTCCTTCCACAAGC | 1) 94 °C x 5 min  2) 94 °C x 45 sec  3) 58 °C x 30 sec  4) 72 °C x 1 min  5) 72 °C x 10 min  steps 2-4 repeated for 35 cycles | Wild-type: ~300 bp  *Gnat1* knockout: ~200 bp |
| Wild-type *Des1*, Floxed *Des1*, ΔE2 *Des1* | LoxPf:  GTGTCACGATGCCCAGGTGTT  E2:  CCTGGGAAAAACCTGCCCATG  FRTr:  ACCGCAATTCCTTATCATGTTGTAT | 1) 94 °C x 5 min  2) 94 °C x 45 sec  3) 58 °C x 30 sec  4) 72 °C x 1 min  5) 72 °C x 10 min  steps 2-4 repeated for 30 cycles | Wild-type:  693 bp  Floxed *Des1*: 815 bp  ΔE2 *Des1*:  543 bp |
| Alb-Cre | 20239:  TGCAAACATCACATGCACAC  20240:  TTGGCCCCTTACCATAACTG  oIMR5374:  GAAGCAGAAGCTTAGGAAGATGG | 1) 94 °C x 5 min  2) 94 °C x 45 sec  3) 55 °C x 30 sec  4) 72 °C x 1 min  5) 72 °C x 10 min  steps 2-4 repeated for 30 cycles | Wild-type:  351 bp  Transgene: 390 bp |
| Pdgfrα-Cre | oIMR1084:  GCGGTCTGGCAGTAAAAACTATC  oIMR1085:  GTGAAACAGCATTGCTGTCACTT | 1) 94 °C x 5 min  2) 94 °C x 45 sec  3) 58 °C x 30 sec  4) 72 °C x 1 min  5) 72 °C x 10 min  steps 2-4 repeated for 35 cycles | Wild-type:  no reaction  Transgene: ~100 bp |

**SUPPLEMENTARY FIGURE LEGENDS**

**Figure S1 –** *Expression of retinal cell type-specific genes within cell clusters identified by t-SNE.* Genes are specified by their Mouse Genome Informatics (MGI) abbreviations. Genes used to identify specific cell clusters include *Rho* (rods), *Opnsw*1 (S-cones), *Pou4f1* (retinal ganglion cells), *Rpe65* (retina pigment epithelium), *Onecut2* (horizontal cells), *Mpeg1* (microglia), *Icam2* (endothelial cells), *Pdgfrα* (astrocytes), and *Kcnj8* (endothelial cells).

**Figure S2 –** *Expression of retinal bipolar cell subtype-specific markers identified by t-SNE.* Genes are specified by their Mouse Genome Informatics (MGI) abbreviations. Genes used to identify specific cell clusters include *Bhlhe23* (rod bipolar cells and a subset of cone bipolar cells), *Prkca* (rod bipolar cells), *Sebox* (subset of cone bipolar cells), *Cabp5* (subset of cone bipolar cells), *Vsx2* (pan-bipolar cell marker) and *Vsx1* (subset of cone bipolar cells).

**Figure S3 –** *Expression of amacrine and bipolar subtype-specific markers within cell clusters identified by t-SNE.* Genes are specified by their Mouse Genome Informatics (MGI) abbreviations. Genes used to identify specific cell clusters include *Gad1* (GABAergic amacrine cells), *Gad2* (GABAergic amacrine cells), *Slc6a9* (glycinergic amacrine cells), *Tfap2b* (pan-amacrine cell marker), *Pax6* (pan-amacrine cell, Müller glia, horizontal cell and retinal ganglion cell marker), *Prox1* (rod bipolar cells and subset of amacrine cells), *Lsl1* (rod bipolar cells and a subset of cone bipolar cells), *Lsl1* (rod bipolar cells, a subset of cone bipolar cells, and a subset of amacrine cells), and *Elavl3* (pan-amacrine cell marker).

**Figure S4 –** *t-SNE analysis of cell clusters from the four biological replicates used in this study.* P60, post-natal day 60.

**Figure S5 –** *Expression of Des1 and genes known to control retinal metabolism within cell clusters identified by t-SNE.* Genes are specified by their Mouse Genome Informatics (MGI) abbreviations.
