# Supplementary figures and images for: Conditional deletion of Des1 in the mouse retina does not impair the visual cycle in cones
Source: FASEB J. 2019 Jan 15;33(4):5782–92. doi: 10.1096/fj.201802493R (PMC6436658; doi:10.1096/fj.201802493R)

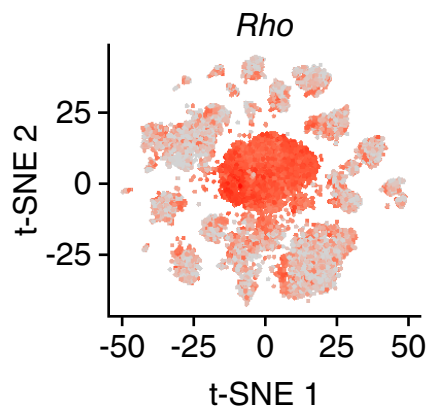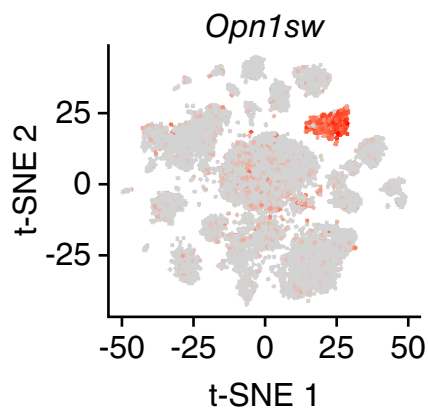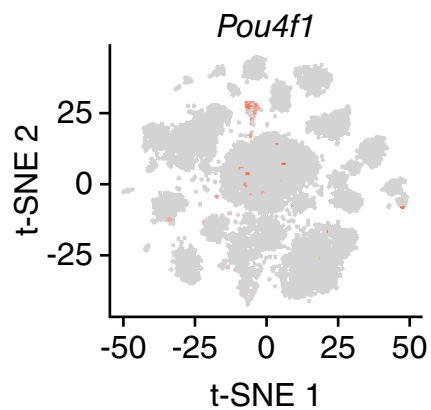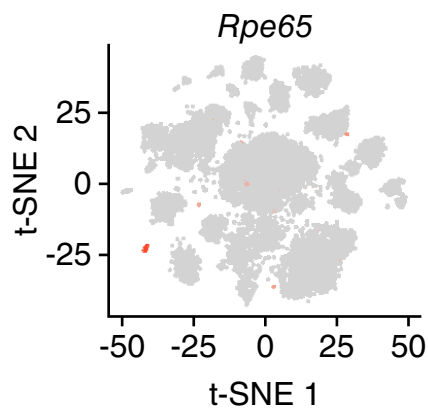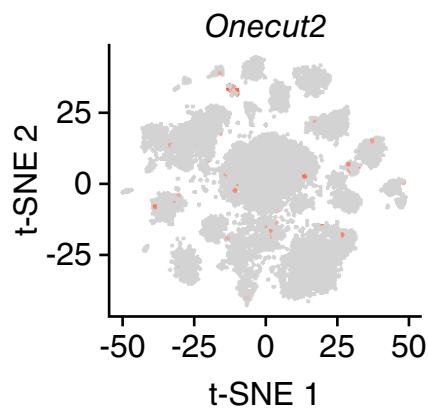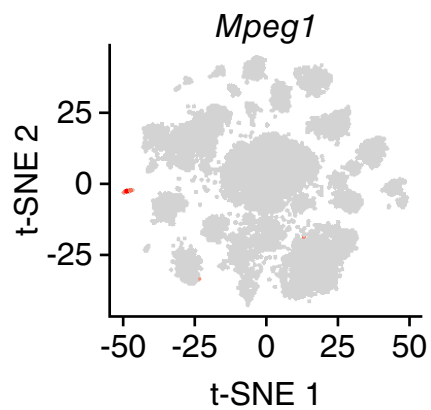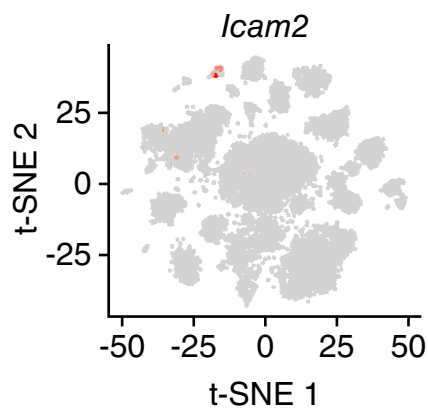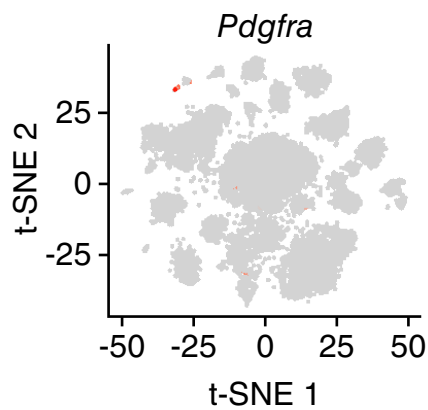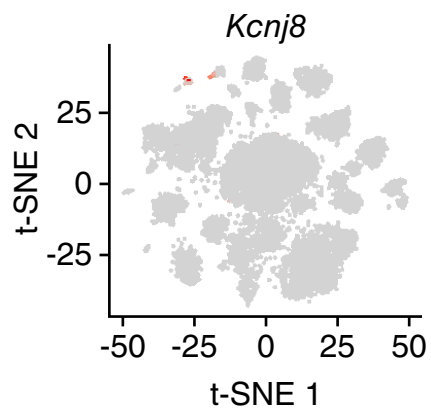

Supplement: Supplementary file 2 [file fj.201802493R.sf1.pdf]

*Bhlhe23*

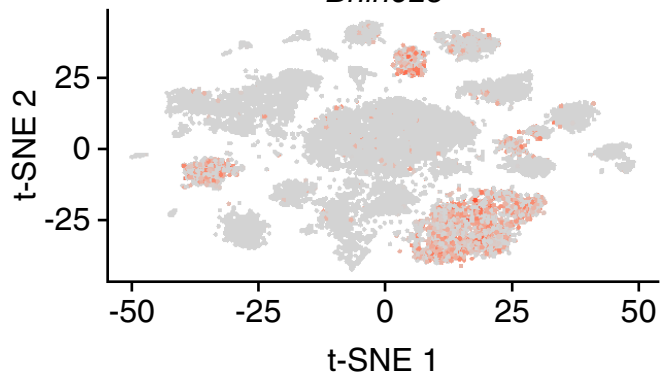

*Prkca*

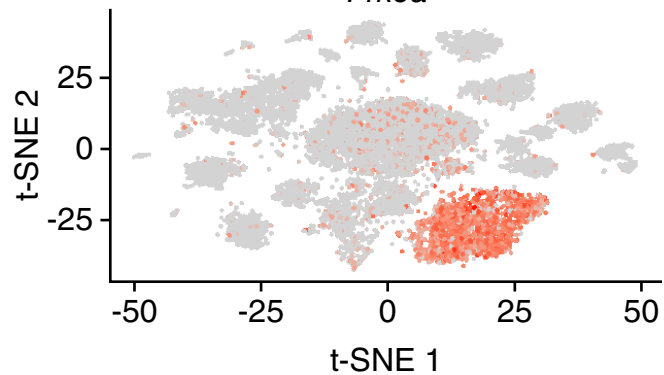

*Sebox*

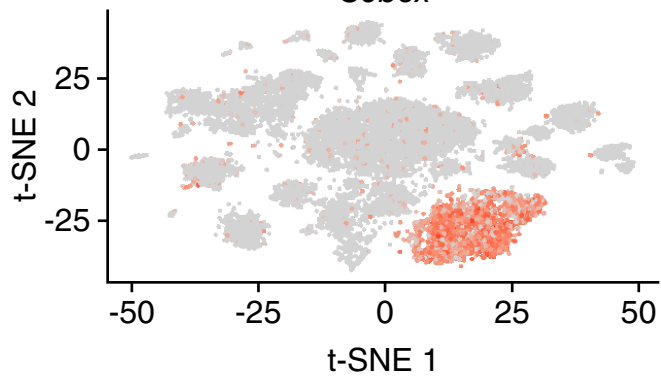

*Cabp5*

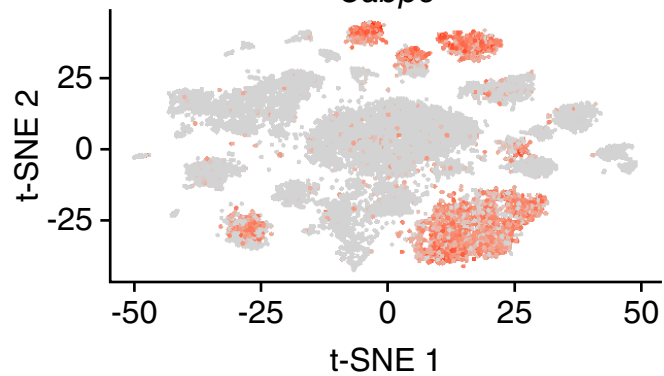

*Vsx2*

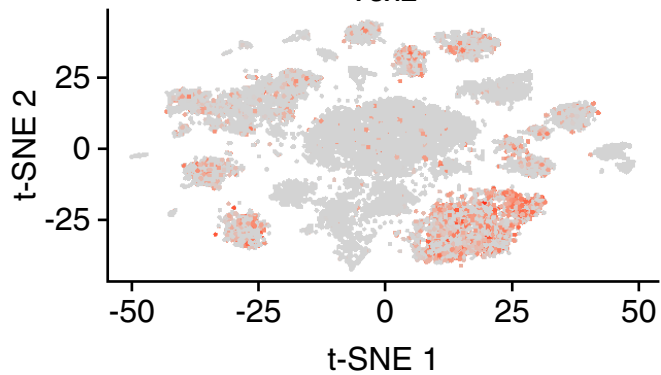

*Vsx1*

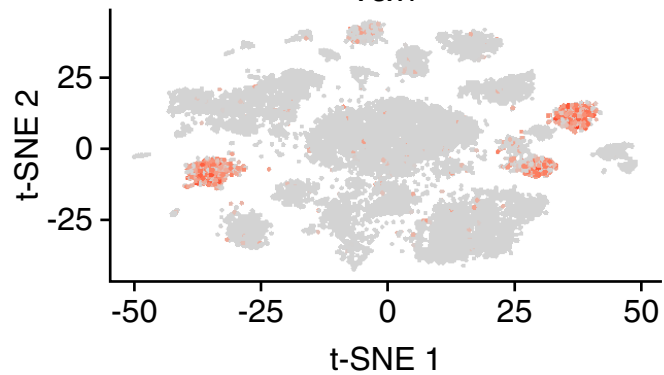

Supplement: Supplementary file 3 [file fj.201802493R.sf2.pdf]

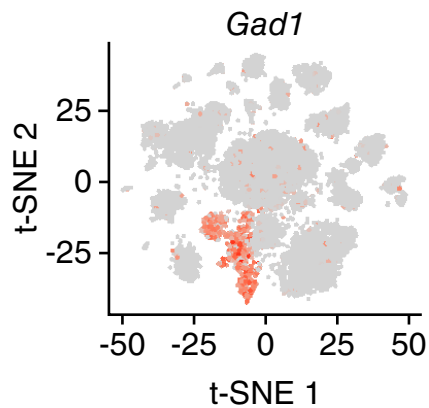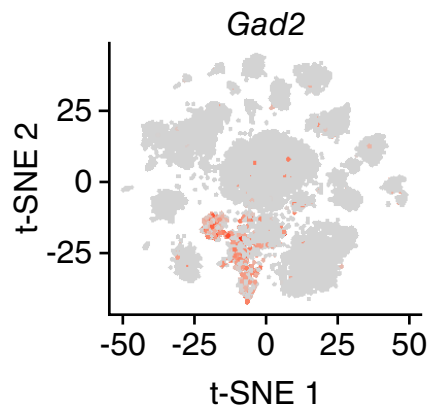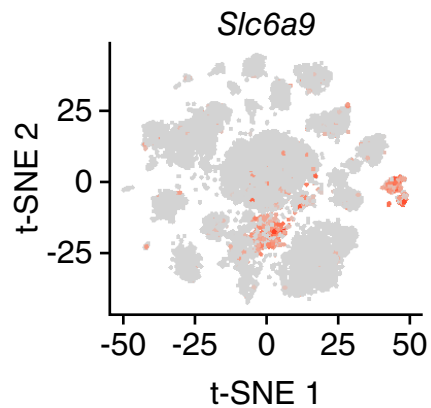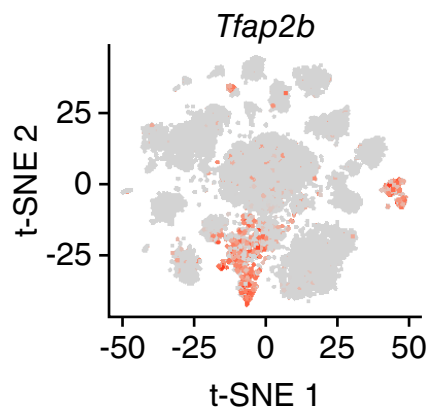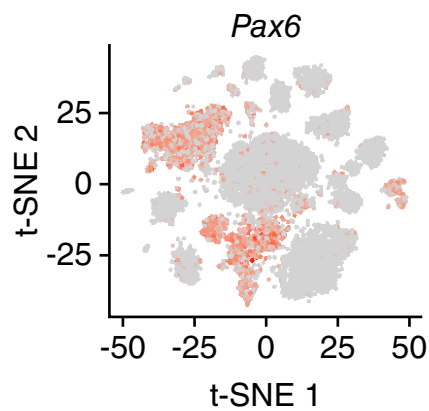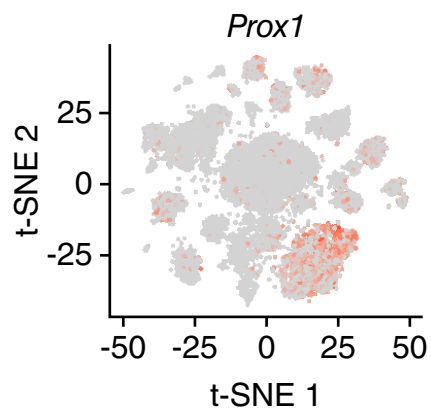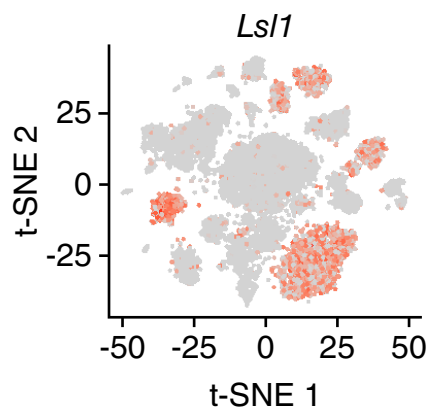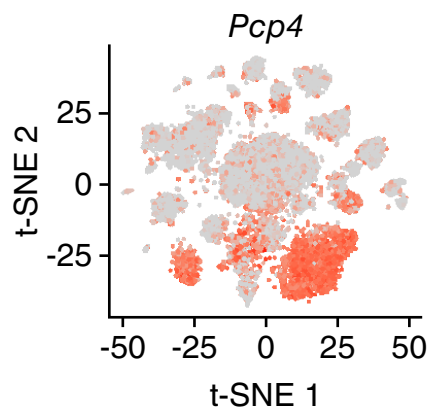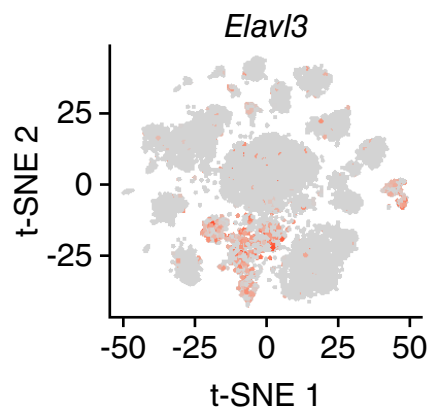

Supplement: Supplementary file 4 [file fj.201802493R.sf3.pdf]

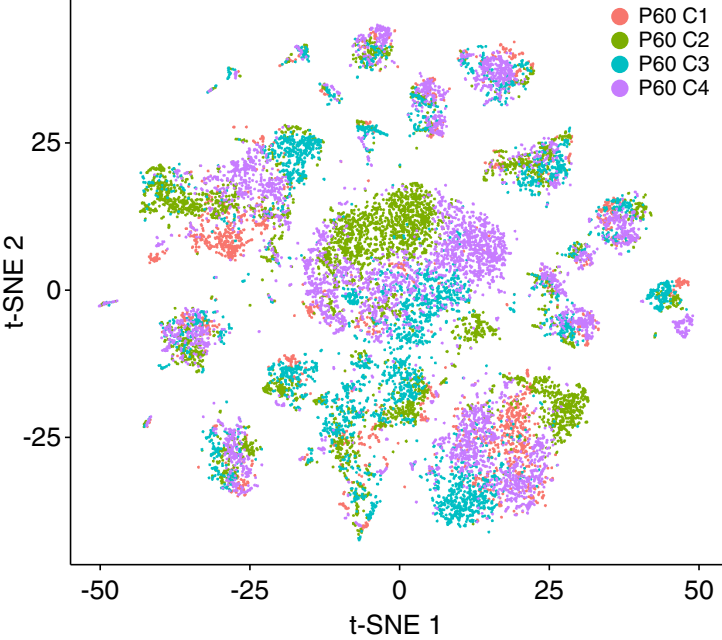

Supplement: Supplementary file 5 [file fj.201802493R.sf4.pdf]

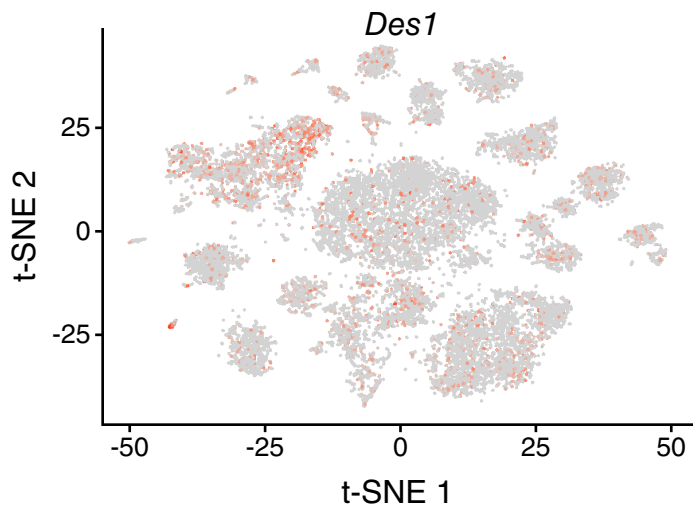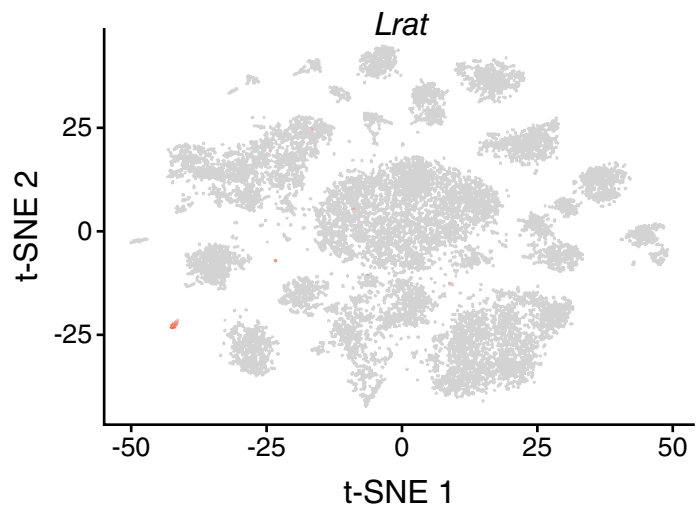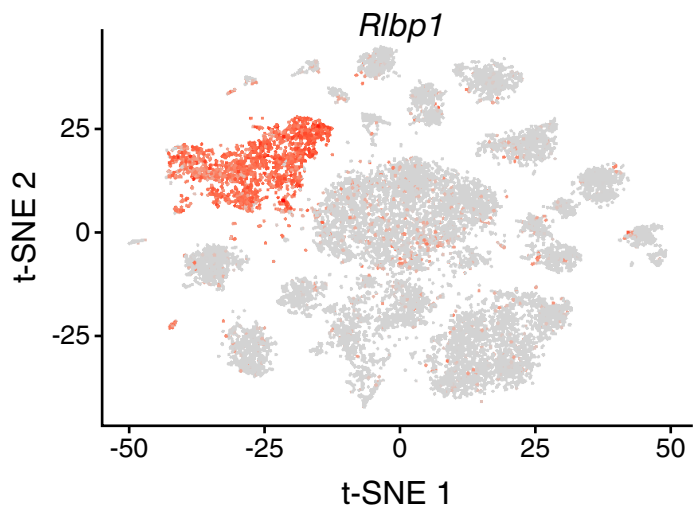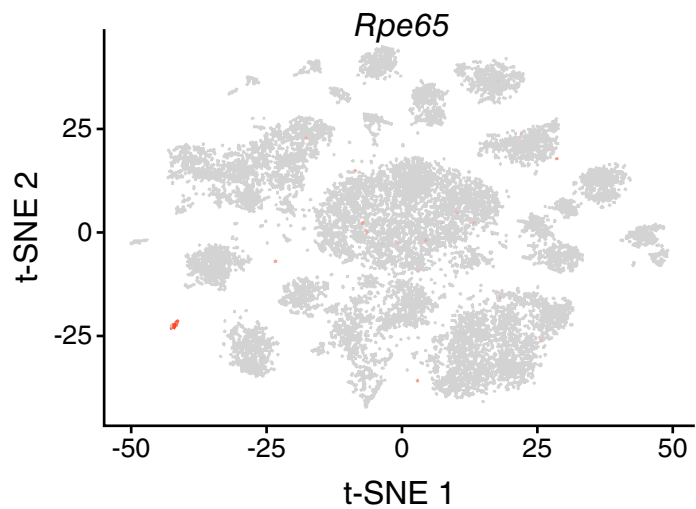

Supplement: Supplementary file 6 [file fj.201802493R.sf5.pdf]
